# Crisis Brings Innovative Strategies: Collaborative Empathic Teleintervention for Children with Disabilities during the COVID-19 Lockdown

**DOI:** 10.3390/ijerph18041749

**Published:** 2021-02-11

**Authors:** Verónica Schiariti, Robin A. McWilliam

**Affiliations:** 1 Division of Medical Sciences, University of Victoria, Victoria, BC V8W 2Y2, Canada; 2 Department of Special Education and Multiple Abilities, The University of Alabama, Tuscaloosa, AL 35405, USA; theramgroup0@gmail.com

**Keywords:** COVID-19, functioning, participation, Routines-Based Model, family-centered, child, pandemic, teleintervention, abilities, rights

## Abstract

*Background:* While coronavirus disease 2019 (COVID-19) continues to spread across the globe, public health strategies—including the social distancing measures that many countries have implemented— have caused disruptions to daily routines. For children with disabilities and their families, such measures mean a lack of access to the resources they usually have through schools and habilitation or rehabilitation services. Health emergencies, like the current COVID-19 pandemic, require innovative strategies to ensure continuity of care. The objective of this perspective paper is to propose the adoption of two innovative strategies for teleintervention. *Methods:* The novel strategies include: (1) to apply the principles of the Routines-Based Model beyond the early years of development, and (2) to adopt My Abilities First—which is a novel educational tool promoting an abilities-oriented approach in healthcare encounters. *Results:* In the context of COVID-19, and using accessible language, the content of the paper highlights what is important for families and individuals with disabilities, and how the proposed novel strategies could be useful delivering remote support. *Conclusions:* The principles of the Routines-Based Model and My Abilities First are universal and facilitate collaborative, empathic, family-centered teleintervention for children and youth with disabilities during and post the COVID-19 lockdown.

## 1. Crisis Brings Innovative Strategies

The coronavirus disease 2019 (COVID-19) is causing a global crisis, not only because of the rapid spread of the virus but also the impact of public health strategies currently in place, aiming to stop human-to-human transmission. The social distancing measures that many countries have implemented have caused huge disruptions to daily routines. Children with or without disabilities are no longer playing outside, as playgrounds are currently closed in most countries and lockdown restricts outings. Families are no longer getting together, celebrating, or sharing key milestones with extended family members, and children are not seeing their grandparents, cousins, or uncles and aunts. Almost 90% of children are not attending schools around the world; that is, 1.5 billon children are not receiving regular education [1]. For children with disabilities and their families, social distancing measures mean a lack of access to the resources they usually have through schools and habilitation or rehabilitation services [2,3]. This shows the need for a change in service provision to ensure continuity of care for children with disabilities. 

During a crisis, like the COVID-19 pandemic, it is essential to prioritize inclusion, collaboration, diversity, and equity. Most important, in our opinion, for the habilitation and rehabilitation of children with disabilities, the COVID-19 crisis may facilitate the global adoption of innovative strategies in remote service provision. Some examples of changes that can be accelerated during and after the COVID-19 pandemic—at a global level—are as follows: setting collaborative and meaningful goals; focusing on abilities; empowering families; emphasizing children’s rights to express their feelings and opinions; fostering emotional and nurturing connections among professionals and families; changing attitudes towards disability; and improving distance service provision by applying early intervention principles beyond the age of five years. 

While the COVID-19 pandemic continues to spread across the globe, children with or without disabilities continue to grow and develop. Children’s development is a dynamic process by which the child moves progressively from dependency on everyday functioning towards maturity and independence. In this dynamic process, the child’s functioning depends on continuous interactions with the family or other caregivers. These interactions frame the acquisition of various skills, showing the importance of seeing the child in the context of the family [4]. For children with disabilities who need habilitation and rehabilitation, service delivery models that base their interventions on the children’s natural contexts to support them in their daily routines and functioning are essential. 

## 2. Proposed Innovative Strategies Using Remote Support

Based on our experience working in the field of early child development and childhood disability in different countries and embracing the global academic solidarity that the COVID-19 crisis has generated, in this paper we share selected innovative strategies to support continuity of care for children with disabilities from birth to transition to adulthood. As such, the objective of this perspective paper is to propose the adoption of two innovative strategies for teleintervention for children and youth with disabilities and their families. Specifically, using remote or virtual support, we propose: (1) to apply the Routines-Based Model (RBM) beyond the early years of development, and (2) to adopt My Abilities First in healthcare encounters. These strategies promote collaborative, empathic, family-centered teleintervention for children with disabilities during and post the COVID-19 lockdown. 

The RBM is a collection of practices that, together, provide a unified approach to working with young children aged 0 to 5 years with disabilities and their families [5]. It emphasizes (a) children’s functioning in their everyday routines and (b) supporting families. The RBM practices are well defined, have implementation checklists, and are supported by research. The model has three main components: needs assessment and intervention planning; a consultative approach; and a method for running classrooms. RBM is based on a simple premise: all the intervention occurs between visits. This premise means that the visit should be based on building the caregiver’s capacity. RBM is implemented in many different cultures, continents, and contexts [5].

My Abilities First is an open access educational tool promoting an abilities-oriented approach to disability evaluation and intervention [6]. This e-tool educates professionals and the general public about the importance of having a positive attitude towards disability, acknowledging that people with disability have the right to attain the highest standard of health care, without discrimination. My Abilities First encourages the systematic identification of strengths and limitations performing everyday routines, as well as barriers and facilitators influencing functioning. 

**Figure 1:**
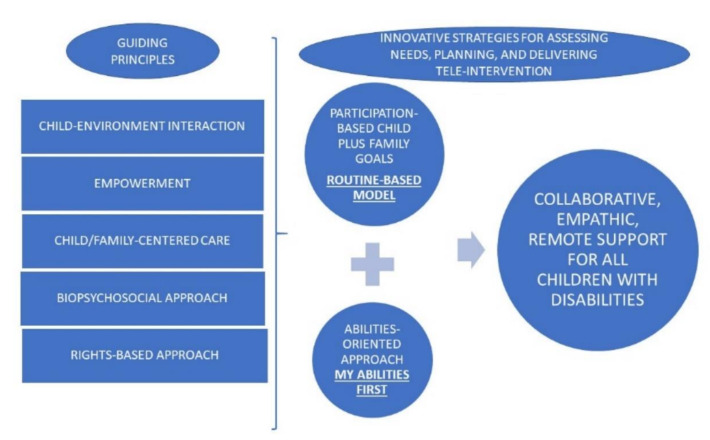
Innovative strategies for delivering remote support for all children with disabilities.

As shown in Figure 1, RBM and My Abilities First share guiding principles for delivering comprehensive services for children with disabilities and their families, including the dynamic role of child–environment interactions, the importance of delivering child/family-centered care, the adoption of a biopsychosocial and a rights-based approach for needs assessments, planning and interventions, and the ultimately goal of empowering families and children to make decisions about their care, among others. 

## 3. The Need for New Service Delivery Strategies during COVID-19

### 3.1. What Is Important?

Firstly, using the principles of RBM, this article addresses what is different during the lockdown, how to assess needs with a conversation, the importance of emphasizing meaningful functioning and participation in a support plan, using family consultation in virtual visits, and the need for professionals to look after themselves. Secondly, this paper introduces the novel My Abilities First and highlights the importance of adopting a rights-based approach during the COVID-19 pandemic. Importantly, this paper invites the readers to reflect on their current practices and consider new strategies for delivering teleconsultation and/or teleintervention, celebrating strengths and abilities in natural environments where children live and grow.

### 3.2. What Is Different during COVID-19?

During the lockdown, professionals who typically work in schools, clinics, or other service delivery environments now find themselves making videoconferencing calls to children and their families. This strange situation allows us to learn lessons from early intervention (birth to five), where professionals have been visiting homes for decades [5,7]. They therefore have methods for working with families in the context of everyday routines. These methods, if done correctly, are largely transferable to videoconference calls. By done correctly, we mean using the visits to build the family’s capacity to “intervene” with their child throughout the week [7]. Incorrect home visits would be professionals working directly with the child for, say, an hour and expecting that tiny dose to be generalized and applied to the rest of the week. The older a child is, the more that kind of tutorial-type visit could work. With young children, it does not. The RBM has numerous strategies that have already been disseminated as applicable in early intervention through virtual visits in several countries [8]. In this paper, the authors propose to expand RBM for families of children with disabilities of all ages.

Virtual visits during the lockdown are different from home visits at other times for a few reasons. First, the adults often get no break: they are forced to be at home with their children with no additional help. Weekends are great but the longest weekend ever is not! Especially vulnerable to this stress are adults who are caring for and entertaining children alone, such as single parents or the partners of essential workers. Similarly, for some siblings, having no time away from their brothers or sisters can be exhausting. This situation is obviously exacerbated if one of the children has challenging behaviors. Second, in many households, privacy is unobtainable. Third, life can be boring at home, with day after day of no change, especially children who have difficulties with attention or independent play or who are simply the products of the modern age can become easily bored—and every parent knows how trying it is to have children who say they are bored. 

The lockdown has created a quite-different environment, but professionals working with children of all ages with disabilities are trying to support children and families through videoconferencing. One place to begin is to reassess needs.

### 3.3. Assess Needs with a Virtual Conversation

During the lockdown, technology is fundamental to carry on everyday life. Everyday life is what this article is about, and technology is our way of connecting with families’ everyday lives.

With children’s and families’ lives disrupted by the lockdown, the functioning needs of the child and the families’ needs are different. We can consider a routines-based conversation, family-level questions, and support the family to choose their goals. 

Professionals can ask families about their day-to-day life, beginning with waking up and all the way to adults’ going to bed. At each time of the day (i.e., routine, as we define it), we ask the family to describe the child’s engagement, independence, and social relationships in that routine. Structuring the conversation by routines is natural, in that families can walk you through their day, and contextual: we hear about children’s functioning, and when and where the skills are needed. We ask families what children are interested in as well as what they can do. When those abilities and interests do not match the demands of the routine, we have diminished functioning. We note these concerns as we are talking to families, but they are not goals. If we have a good, in depth conversation we will uncover many such concerns—more than needed for our goal plan—and the Routines-Based Model is notorious for already having long lists of goals. The routines-based conversation is based on the well-known, evidence-based Routines-Based Interview (RBI) [9,10,11]. During this Routines-Based Conversation, if the child in question is old enough to participate meaningfully, this contribution can add to the richness of the discussion. The family should make the determination whether the child’s participation would be helpful or disruptive. One of the tenets of the RBM is the question “Whose child is it?”.

In addition to the conversation about child functioning in routines, the conversation can include the time, worry, and change questions, a hallmark of the RBI: do you have enough time for yourself or yourself and another person? When you lie awake at night, worrying, what do you worry about? If you could change anything in your life, what would it be?

These powerful questions have two benefits. First, they show the family that you care enough to ask these questions about the adults’ well-being. You can also ask them of the child, if appropriate. Second, they go beyond daily routines to deeper, more emotional needs, which is precisely why some professionals try to avoid them—professionals who are not used to dealing with emotions or who mistakenly think that they need to fix any need mentioned. As we said earlier, families at this point are not choosing goals.

After asking the time, worry, and change questions, we might recap the conversation so far, in just a few minutes, to remind the family of the concerns. This is a reminder: it is not a time to rehash the whole discussion or to try to make the family feel good about their routines. The purpose of a recap is to remind them of concerns.

### 3.4. Set Goals Addressing Meaningful Participation

The family then chooses goals for their child and themselves. The child can participate in this process, as appropriate, and the family has editing power, meaning the family has the last say in what goals are decided upon. The professional can help the family by looking at the notes and reminding the family of concerns that arose during the routines-based conversation. Typically, the RBI produces 10–12 goals, both child and family goals. The routines-based conversation might produce fewer but should still be six or more goals.

The professional reads or shows, perhaps through a shared screen, the goals the family has chosen and asks them to put these into order of importance. If appropriate, the child can participate in this process. Once the professional has this importance prioritization, he or she can create a Goal x Routine matrix, such as shown in the RBM resource page (Access to RBM resource page available here http://eieio.ua.edu/uploads/1/1/0/1/110192129/intervention_matrix_completed_english.pdf (accessed on 2 April 2020)).

Once we know the child’s and family’s needs, we should develop a plan. Research has shown that having goals is better than not having goals [12]. Next, we discuss the importance of child goals addressing meaningful participation (Access to RBM resources provided in Supplementary Material S1).

### 3.5. Emphasize Meaningful Participation in a Plan

Participation has come to be acknowledged as a critical dimension in the definition of disability [4]. The impairment in a person is only as handicapping to that person as it affects the person’s ability to function meaningfully (i.e., participate) in his or her environment [4,13]. We therefore take needs the family identifies and develop them into participation-based goals and family goals.

Participation-based goals put the emphasis or purpose of a skill the child needs to achieve on engagement in a routine. For example, if a parent says she/he wants the child to use two-word combinations, in the RBM, this would have come from a discussion about a need in one or more routines. A goal like this could be for a young child or an older child with significant communication delays. An important point is that the need came from a need for meaningful participation in a routine. Needs in routines should be authentic. 

A participation-based goal might therefore read as “Javier will participate in breakfast time, going outside time, and hanging out time by using two-word combinations.” This goal tells us the skill, using two-word combinations, and the purpose: to participate in those three routines. It is a perfect participation-based goal but it is not measurable. When professionals want goals to be measurable, they can keep the goal and add acquisition, generalization, and maintenance criteria, as in “We will know he can do this when he uses three two-word combinations at breakfast time, going outside time, and one hanging-out time in one day for four consecutive days.” The acquisition criterion is the frequency of two-word combinations: three. The generalization criterion is the routines: three of them. The maintenance criterion is amount of time: four consecutive days.

In addition to child goals, the list of goals must include at least one family goal. Family goals are necessary because of what, in the RBM, we call the two-bucket principle: a mother can fill her child’s bucket only to the extent her bucket is full. Some family goals are related to the child, such as getting information about the child’s disability. Other family goals are not directly related to the child, such as time for parents alone or fulfilling individual dreams. 

Planning is important, and then professionals help families with that plan. The next section addresses how to help families tackle that plan.

### 3.6. Use Family Consultation in Virtual Visits

How do you help families confined with their children with special needs? If you have been using the RBM, this process is not a big challenge, because the RBM is all about building families’ capacities to meet child and family needs: it is not about direct, hands-on work. This section describes how the virtual visit is focused on a plan, develops strategies with caregivers, and involves three types of action.

The videoconference visit is focused on the previous visit and on goals. By the end of the previous visit, the professional had documented what the parents had decided to try, as strategies, with their child. For older children, it might be what the child was going to try doing, with or without assistance. Families, including the child, if appropriate, had also determined what they wanted the focus of this visit to be. 

Therefore, the professional would know what the topic was. Families should also be given the opportunity, however, to determine the agenda for the visit, so professionals should ask two questions: how have things been going (a general question)? Has anything new happened since our last talk?

These questions allow the family to bring up topics that might not have been previously planned. If the family has something they want to talk about, that sets at least the beginning of the agenda. If they do not, the professional reminds the family about (a) strategies they were going to work on since the last meeting and (b) what the family said they wanted the focus of this visit to be on. 

When discussing what the family had been doing, they reflect on how the intervention has been going. This discussion might lead to the family and the professional tweaking the strategy. The amount of context the professional had had previously would predict how many questions he or she would ask during this strategy tweaking: if the professional had much context, he or she might not need to ask many questions. If the professional did not have much context, he or she should ask many questions to ensure his or her suggestions were relevant. Some children with disabilities are trying strategies themselves, but they are still functioning in home contexts, during this quarantine time. So, families are still integrally involved in the execution of these strategies.

Professionals’ suggestions are the result of finding solutions or problem solving. Traditionally, professionals made recommendations to families quickly, based on good will and experience: Most of us professionals are quick to come up with helpful suggestions for families (Example of professional recommendations provided here https://naturalenvironments.blogspot.com/2014/07/self-regulation-in-working-with-families.html (accessed on 4 April 2020)). We should ask questions before making suggestions, and this process can involve the child, if appropriate (Access to RBM resources provided in Supplementary Material S1).

While determining solutions (i.e., developing strategies) we can incorporate, in virtual visits, three types of action. First, we can see what the child does. The family can show us what the child typically does or is now doing. In addition, the child might say, “Look what I can do”, or the professional might ask the child to show what he or she is doing in that routine. Second, the family can show what they are doing or are considering doing. For example, the family might say, “Look what I’ve been trying to do when I take Ted to the grocery store”, and they show a video clip of a trip to the grocery store. Third, the professional describes a strategy, after asking at least four questions. The professional can be as explicit as possible about the idea and can even demonstrate with a doll. Importantly in the RBM, we always check in with the family with two questions, especially important online: Do you think this strategy will work? As busy as you are at these times of the day, do you think you’ll be able to carry out these strategies you’ve chosen?

Before hanging up, the professional and family, including the child, if appropriate, review what was discussed on this virtual visit, review what the family is going to do between this visit and the next, and the family says what they want the focus of the next virtual visit to be. At this point, the professional can use a Goals x Routines matrix to remind the family of the goals on the plan. In the RBM this information is recorded on the Next-Steps Form (NSF).

We have just discussed two pieces of paper, the NSF and the matrix. When visits are through videoconferencing, the professional and the family will need to work out the best method of seeing this paperwork and other materials. If they are using Zoom or Skype, they can share screens. They might take screen shots, scans, or photographs and send them via email or other social media. 

Family consultation, therefore, can work well in a virtual visit, focusing on the family’s agenda and on goals they have selected. Nevertheless, supporting families virtually can be stressful and exhausting. Hence, the following section, address the importance of looking after yourself.

### 3.7. Look after Yourself

Professionals themselves are on lockdown, so they might have their own children at home, who they are caring for and helping with school work. They are in virtual visits with families, hearing about their difficulties and worries. Many families’ struggles are not about things professionals can necessarily help with. Family-centered professionals can problem solve (solution find, in RBM language) with families, but families might, especially now, just need someone to listen—and listening to difficult situations can be draining.

It is important, therefore, for professionals to keep their expectations appropriate. They should approach each virtual visit with a goal of providing encouragement, a listening ear, and some information, not of solving a problem. 

The NSF can help professionals feel a sense of control over what sometimes seems a chaotic way of supporting families. If you know you are going to have to write down what we did today, you will record and reflect on the major topics of your virtual visit. Furthermore, the commitment the family makes, even if it is only one strategy—maybe even one they are continuing—again helps you feel the visit was productive. Finally, with a few or many visits a day, a professional should tell someone about a success, preferably the professional’s success, not simply the child’s or family’s. You could tell a family member, a friend, a colleague, or a supervisor. With a supervisor, you could say, “I’m not seeking advice or help. I simply want you to hear about a success I had because you understand my work.” Looking after yourself by keeping expectations appropriate, using the NSF, and telling someone about a success will help you keep up your strength to help families.

Professionals might also need to engage in self-care: What do you do for yourself, to refill your bucket and to stave away the anxiety of the lockdown, the coronavirus, the changed home environment, and the problems of families? If you cannot think of something healthy to do for yourself, find something. One of us has taken to walking for an hour up and down hills in the woods every morning. What you do for yourself does not have to be about work. Can you say to yourself, “You are a great home/virtual visitor, this is hard, and you are a caring person”? We are all different and what fills our bucket differs, but this is a time where we need to be aware of our own needs and whether we are meeting those needs.

### 3.8. Focus on “My Abilities First”

The COVID-19 pandemic might accelerate a global change towards inclusive and empathic healthcare encounters, using telemedicine or in person. A change about views of disability and how healthcare services are provided is needed globally [14,15]. Individuals experiencing disabilities have often voiced their unsatisfying and emotionally detached interactions with healthcare professionals [14]. 

A recently created electronic tool—called My Abilities First—breaks with the traditional view of disability and educates about the experience of living with a disability [6]. The rationale for the need of developing this novel abilities-oriented approach tool was based on lessons learned from a qualitative study that sought children’s opinions about their strengths and limitations in performing day-to-day activities [16]. Overall, children and youth with disabilities focused primarily on their abilities and strengths, cheering age-appropriate interests [16]. As a result, My Abilities First was created allowing children and youth with disabilities to use their own words to describe themselves and their functional needs. Moreover, My Abilities First operationalizes a rights-based approach in medical education and practice, emphasizing that users’ opinions about themselves and their needs should be routinely sought [17]. 

My Abilities First can be accessed from mobile devices and it can be used in telehealth during or post the COVID-19 lockdown. Information gathered using this electronic tool can start a conversation with children and adults with disabilities about their needs for interventions or teleintervention. 

My Abilities First could be used along the RBM strategies with families of all children with disabilities, regardless the age or underlying health condition. The web-based animations included in My Abilities First educate healthcare students, professionals, and the general public, changing common societal, incorrect assumptions about disability [16,18], thus enhancing the global impact of using a positive language in healthcare. As such, the global adoption of My Abilities First—during and post COVID-19 lockdown—could bring shared purpose, proximity, empowerment, trust, and joy in healthcare. 

Specifically, My Abilities First consists of three web-based animations [6] (Access to animations provided in Supplementary Material S1). The first video introduces how to apply an abilities-oriented approach in healthcare encounters. It proposes the creation of a “my abilities identification card” which can be included in every health record. The target audience of this video includes health and health- allied professionals (My Abilities identification card animation available here https://youtu.be/WyW6ey3kHvM (accessed on 24 April 2020)).

The second web-animation included in My Abilities First describes the personal experience of a person living with a chronic health condition during healthcare encounters. This animation highlights the importance of applying a holistic approach in routine healthcare encounters, asking questions to identify the strengths of the person and capabilities performing daily routines. The audience of this video includes clinicians, researchers, educators, administrators, and students (My Abilities First—Getting to know me animation available here https://youtu.be/Dnn_-0IEe_Q (accessed on 1 May2020)).

Finally, the third video promotes a change in attitudes towards disability from a child’s perspective. In this animation, a typical developing child advocates for social inclusion of children with disabilities, illustrating the importance of focusing on abilities and changing societal attitudes towards disability. The target audience of this video is the general public, school-aged children, and peers (My Abilities First for peers animation available here https://youtu.be/myHFKggNeGc (accessed on 1 May 2020)). 

## 4. Global Impact of Routines-Based Model and My Abilities First

RBM has been implemented in 10 countries over the past 30 years. The model provides actual practices for implementing a family-centered approach in person or virtually [5,8,19]. The model has been modified to include cultural adaptations in different countries and RBM tools are available in languages other than English. 

Professionals applying RBM reported improved goals, in terms of functionality and measurability, improved team functioning, and more collaborative consultation [9,20]. Moreover, the short and long-term effectiveness of the RBM collaborative interventions has been shown in randomized controlled trials [21,22,23]. 

On the other hand, My Abilities First was recently created; it was published in April 2020 [6]. Since its publication, the e-tool has received global attention; it has been downloaded more than 800 times from all continents. It is currently being translated into Spanish, Portuguese, Chinese, and Polish. A pilot study checking satisfaction and change in healthcare attitudes using My Abilities First is underway in Taiwan (led by Professor Hua-Fang Liao, from the National Taiwan University) and in Brazil (led by Professor Egmar Longo, from the Federal University of Rio Grande do Norte). Furthermore, My Abilities First has been included in the pool of innovative resources listed in the International Alliance of Academies of Childhood Disability (IAACD) COVID-19 Task Force page (IAACD COVID-19 Task Force page https://iaacd.net/iaacd-covid-19-task-force/ (accessed on 5 July 2020)). This international initiative is collecting and sharing information related to the impact of COVID-19 on people with disabilities around the world. Finally, My Abilities First has been selected as the theme of a global campaign launched by the European Academy of Childhood Disability (EACD), inviting children and young adults with disabilities—from around the globe—to share their abilities and major facilitators influencing optimal functioning. This campaign is part of the Global Partnership Day (Global Partnership Day, EACD, My Abilities First https://eacd2021.com/my-abilities-first/ (accessed on 30 January 2021)) organized by the EACD in 2021. 

### Implementation Challenges

The implementation of new strategies can be exciting, but it involves change. Due to the current restrictions related to COVID-19, children with disabilities, their families, and professionals had to accommodate to different ways of receiving and delivering services. Accommodating to new ways of doing things is inherently difficult. Implementation challenges related to RBM have been well-described [20], for example changing professionals’ service delivery approach from a medical model to a truly family-centered approach, where families make meaningful decisions about goals, what to work on in between visits and what to focus on. Implementation challenges related to My Abilities First include barriers to change in practice at the organizational or individual level, as adopting an abilities-oriented approach—in person or virtually—means a different way to collect information, plan and deliver interventions in healthcare encounters. Education highlighting the importance of adopting new approaches based on rights, child and family-centered care is essential to ameliorate these implementation challenges.

## 5. Conclusions

The COVID-19 lockdown has changed our personal and professional routines. This historical event has united the globe. We believe this is a good time to expand good practices (i.e., RBM beyond early intervention, for older children with disabilities), to adopt innovative tools (i.e., routine remote teleintervention for vulnerable populations globally), to change attitudes towards disability and adopt a universal rights-based approach in healthcare encounters with children and adults with disabilities (i.e., adopting My Abilities First, combined RBM teleintervention with My Abilities First). 

In summary, it is possible to leverage the COVID-19 crisis by adopting innovative strategies in the field of childhood disability, like RBM teleintervention for all children with disabilities beyond the early years of life, and by facilitating access to the universal inclusive educational tools discussed in this paper. 

## Data Availability

Data sharing is not applicable to this article as this is an opinion paper.

## Supplementary Materials

The following are available online at https://www.mdpi.com/1660-4601/18/4/1749/s1, Routine Based Model (RBM) Intervention Matrix; RBM professional recommendations; links to My Abilities First web animations; IAACD COVID-19 Task Force page; 33rd EACD annual meeting—Global Partnership Day page—My Abilities First campaign.
