# Supplementary material for: Crisis Brings Innovative Strategies: Collaborative Empathic Teleintervention for Children with Disabilities during the COVID-19 Lockdown
Source: Int J Environ Res Public Health. 2021 Feb 11;18(4):1749. doi: 10.3390/ijerph18041749 (PMC7916822; doi:10.3390/ijerph18041749)
Supplement: Supplementary file 1 [file ijerph-18-01749-s001.pdf]

Routine-based model (RBM) resource

EIEIO
Evidence-based International
Early Intervention Office

INTERVENTION MATRIX (COMPLETED)

	Routines							
Goals/Outcomes	Dressing	Meals	Outside	Play	Before Dinner	Bath	Bedtime	
1. Persist	X	X		X				
2. Follow 2-step directions				X	X		X	
3. Mother resume education								
4. Uses 3 different pieces of playground equipment			X					
5. Use fork with little spilling		X						
6. Parents info about Fragile X syndrome								
7. Wait for others' turns				X				
8. Put on shirt and pants	X					X		
9. Parents time for themselves								
10. Count to four when upset		X		X			X	
11. Express opinion without whining or crying				X	X		X	
[illegible]								

Access full page here
http://eieio.ua.edu/uploads/1/1/0/1/110192129/intervention_matrix_completed_english.pdf

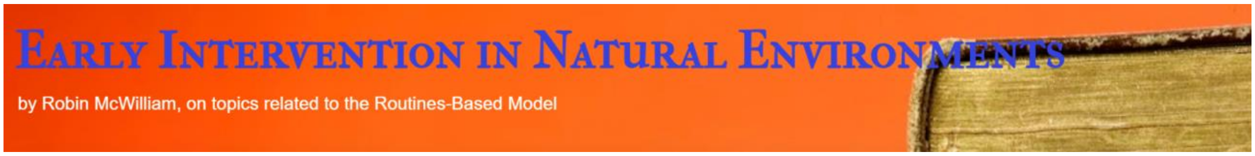

Example of RBM professional recommendations

Self-Regulation in Working With Families
Home-based early interventionists need self-regulation too. In this post I discuss what relationship we have with families, who the hero of the visit is, and the Hoosiers Rule.

As we develop our relationship with families, do we present ourselves as the people with answers? To some extent, we do, to build the family's confidence in us and to show them they're not wasting their time hosting us. But if we always have the answers, what are we doing to families? We might be enhancing their dependence on us rather than their self-confidence as parents, problem solvers, and "case managers" for their child. As Anne Isabella Thackeray

We need to be careful not to try to be the hero of the visit, which can happen if we have all the answers—the strategies, the ideas, the suggestions, the information…. Getting into the car at the end of the visit, when we have given the family the gift of our creativity, wisdom, and knowledge, we feel self-satisfied. We feel useful. We are immediately gratified. This feeling is addictive. Many home visitors are absolutely convinced their role is to give families everything they can, right away. They are convinced because families love them and they themselves have that feeling of self-satisfaction at the end of home visits.

Hold off on that brilliant idea and work *with* the family.

- · Tell me more about the problem.
- · What time of the day does this happen, how often, for how long?
- · What would you like to see happen instead?
- · What have you already tried and what happened?
- · Have you ever tried _____?

To each of these questions, there could be many follow-up questions, so the discussion is often detailed and of some length. The last question, which I have dubbed, in Routines-Based Early Intervention, “ask-to-suggest,” is actually a suggestion. It might not be the strategy the family implements, because they might not think it’s feasible, but “Have you tried _____?” should not be considered a lead-up question: It’s really a suggestion in the form of a question.

This approach to working with families, which we call **family consultation**, builds families' skills in arriving at solutions, makes them feel like a partner not a client, and might even make them feel like the hero of the visit.
Read full Post by Robin McWilliam here [Early Intervention in Natural Environments]

MY ABILIIES FIRST: positive language in health care, Schiariti V.

The first video introduces how to apply an abilities-oriented approach in healthcare encounters. It proposes the creation of a “my abilities identification card” which can be included in every health record. The target audience of this video includes health and health- allied professionals
Video 1. Introducing the ‘my abilities’ ID card (target audience: health and health allied professioals). Access animation here https://youtu.be/WyW6ey3kHvM

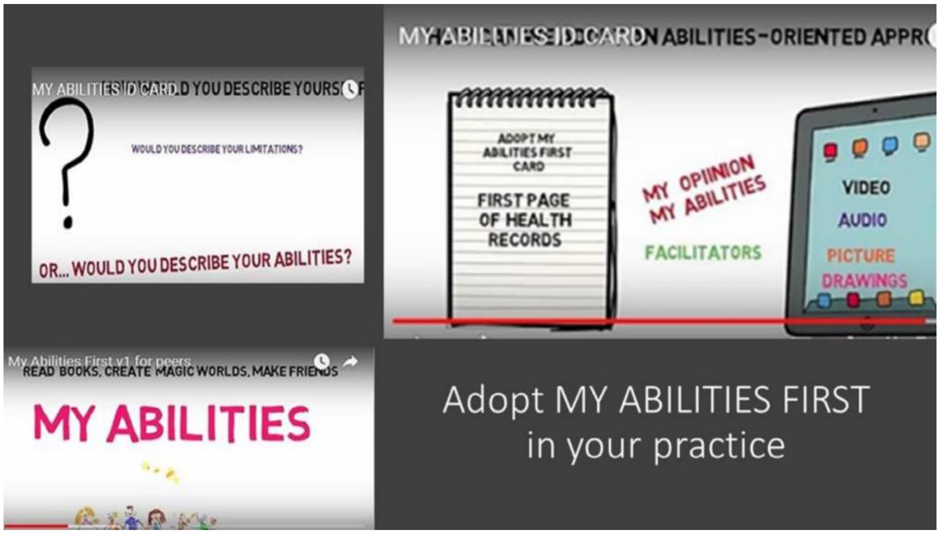

The second web-animation included in My Abilities First describes the personal experience of a person living with a chronic health condition during healthcare encounters. The audience of this video includes clinicians, researchers, educators, administrators, and students

Video 2. Promoting an abilities-oriented approach (target audience: clinicians,researchers, educators, administrators and students): Access animation here
https://youtu.be/Dnn_-0IEe_Q

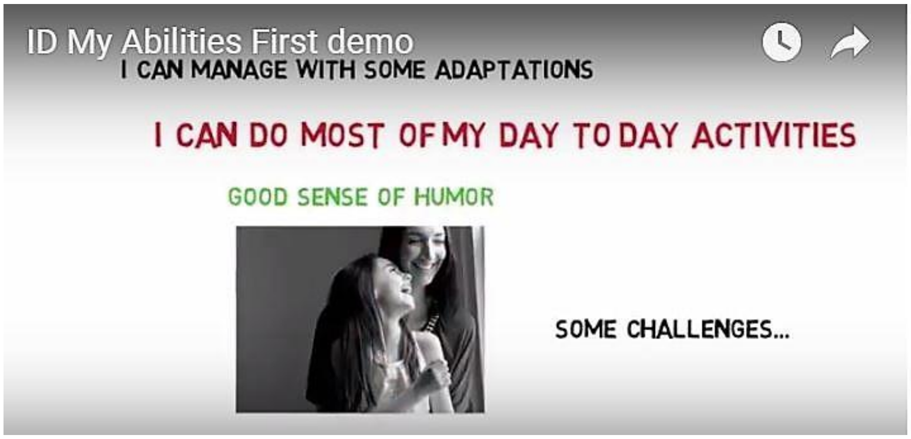

The third video promotes a change in attitudes towards disability from a child's perspective. In this animation, a typical developing child advocates for social inclusion of children with disabilities, illustrating the importance of focusing on abilities and changing societal attitudes towards disability. The target audience of this video is the general public, school-aged children, and peers

Video 3. Changing societal attitudes: Access animation here https://youtu.be/myHFKggNeGc

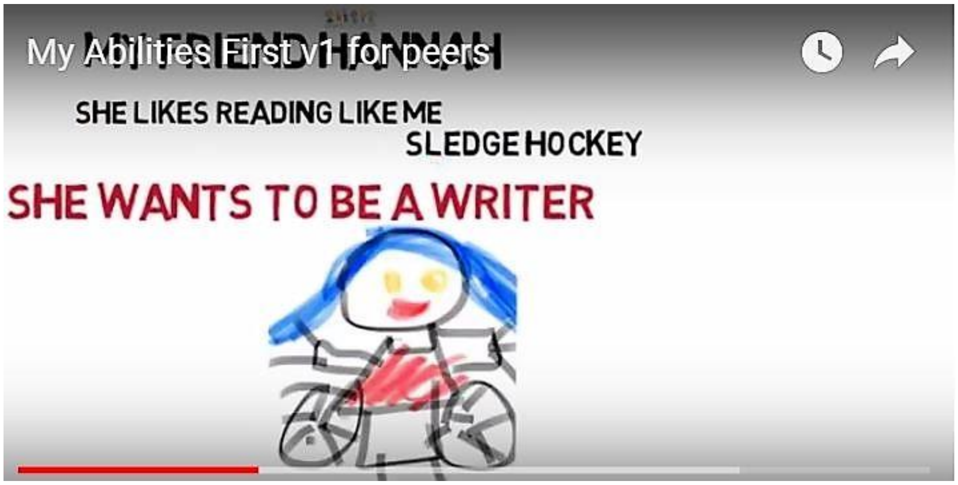

Read full paper here MY ABILITIES FIRST: Positive language in health care - Schiariti - 2020 - The Clinical Teacher - Wiley Online Library

IAACD > • IAACD COVID-19 Task Force

IAACD COVID-19 Task Force

Welcome to IAACD COVID-19 Task Force page!

Successful ideas/strategies used during COVID-19

Here you can find resources that are describing successful ideas/strategies used during COVID-19 lockdown and reopening phase.

My Abilities ID Card

During and post COVID-19 MY ABILITIES FIRST consists of short animated videos promoting an abilities-oriented approach in every encounter with a child or adult experiencing a disability. MY ABILITIES FIRST can be accessed from mobile devices and it can be used in tele-health during the COVID-19 lockdown The web-based animations included in MY ABILITIES FIRST educate healthcare students, professionals, and the general public, changing common societal, incorrect assumptions about disability. Importantly, MY ABILITIES FIRST promotes a rights-based approach during and post COVID-19.

Author: MD Veronica Schiariti

Watch video.

Access full materials on the IAACD COVID-19 TASK FORCE PAGE here COVID-19 Successful Ideas – IAACD

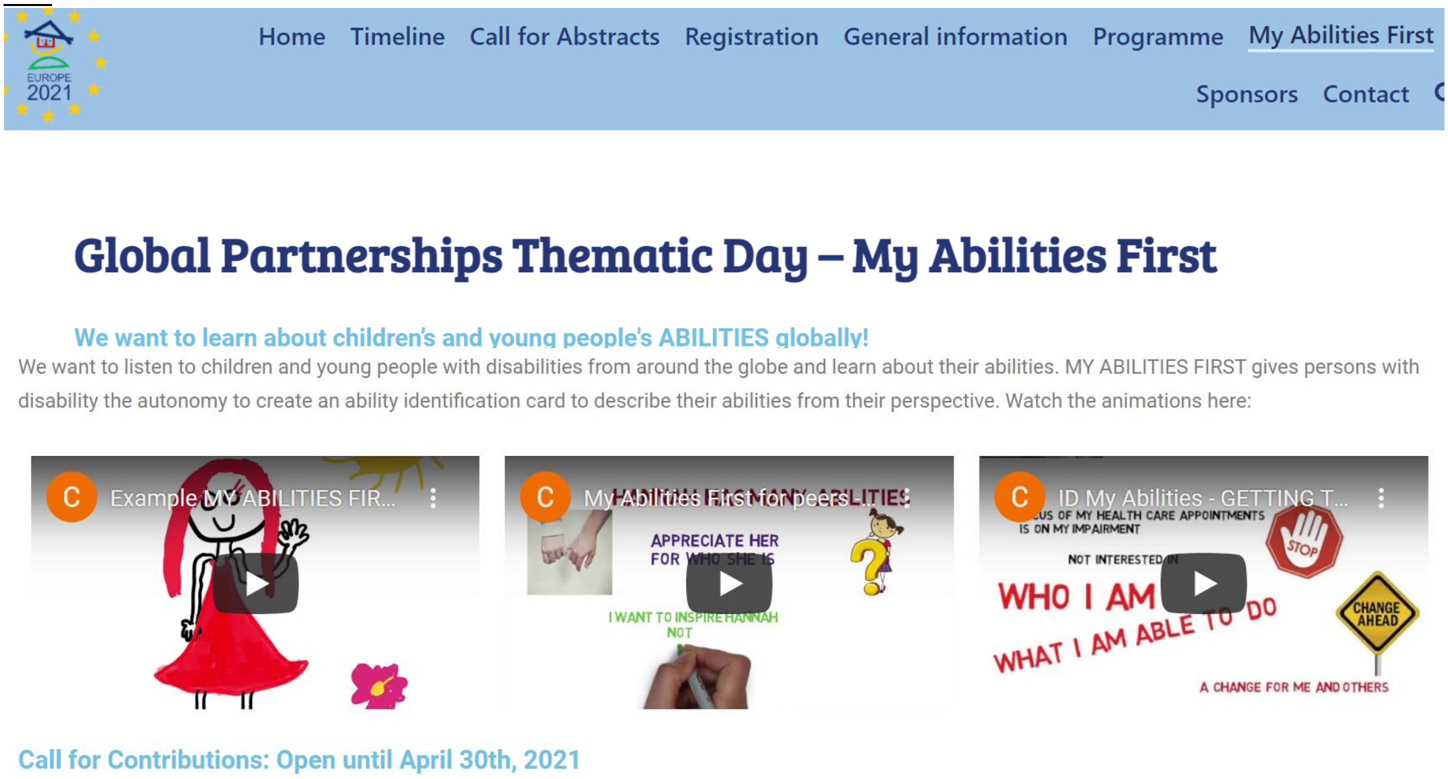

Access the European Academy of Childhood Disability 2021 - 33rd annual meeting here
[My Abilities First – EACD 2021 – 33rd Annual EACD Meeting]
